# Supplementary material for: Complete Genomic Characterisation and Mutation Patterns of Iraqi SARS-CoV-2 Isolates
Source: Diagnostics (Basel). 2022 Dec 21;13(1):8. doi: 10.3390/diagnostics13010008 (PMC9818665; doi:10.3390/diagnostics13010008)
Supplement: Supplementary file 1 [file diagnostics-13-00008-s001.zip › diagnostics-2082863-supplementary.pdf]

Supplementary Table S1. Full genomic annotations of 10 Duhok isolates from the third wave of the SARS-CoV-2 pandemic

Genomic region	Nucleotide variation	AA change	Isolates	Exonic Function	Mutation per region	% of tatotal muattions
5'UTR	G210T	NA	all 10 samples	Upstream	3	2.941176471
5'UTR	C241T	NA	all 10 samples	Upstream		
5'UTR	G242T	NA	6	Upstream		
ORF1a;ORF1ab;nsp1	NA	NA	NA	NA	0	0
ORF1a;ORF1ab;nsp2	C2062T	**A419A**	4	synonymous SNV	2	1.960784314
ORF1a;ORF1ab;nsp2	C2094T	**S430L**	1	nonsynonymous SNV		
ORF1a;ORF1ab;nsp3	C3037T	**F106F**	all 10 samples	synonymous SNV	17	16.66666667
ORF1a;ORF1ab;nsp3	C3096T	**S126L**	5	nonsynonymous SNV		
ORF1a;ORF1ab;nsp3	C3099A	**T127N**	7	nonsynonymous SNV		
ORF1a;ORF1ab;nsp3	G3320A	**V201I**	3	nonsynonymous SNV		
ORF1a;ORF1ab;nsp3	G4181T	**A488S**	1,2,3,4,5,6,7,10	nonsynonymous SNV		
ORF1a;ORF1ab;nsp3	C4575T	**T619I**	3	nonsynonymous SNV		
ORF1a;ORF1ab;nsp3	T4888C	**P723P**	8,9	synonymous SNV		
ORF1a;ORF1ab;nsp3	G5062A	**L781L**	6	synonymous SNV		
ORF1a;ORF1ab;nsp3	C5184T	**P822L**	8,9	nonsynonymous SNV		
ORF1a;ORF1ab;nsp3	A5462C	**S915R**	2,5	nonsynonymous SNV		
ORF1a;ORF1ab;nsp3	A5584G	**T955T**	8,9	synonymous SNV		
ORF1a;ORF1ab;nsp3	T5653C	**Y978Y**	6	synonymous SNV		
ORF1a;ORF1ab;nsp3	C6402T	**P1228L**	1,2,3,4,5,6,7,10	nonsynonymous SNV		
ORF1a;ORF1ab;nsp3	C7124T	**P1469S**	1,2,3,4,5,6,7,10	nonsynonymous SNV		
ORF1a;ORF1ab;nsp3	T7657G	**F1646L**	9	nonsynonymous SNV		
ORF1a;ORF1ab;nsp3	A7886G	**I1723V**	8,9	nonsynonymous SNV		
ORF1a;ORF1ab;nsp3	C7955T	**L1746F**	3	nonsynonymous SNV		
ORF1a;ORF1ab;nsp4	T8737C	**D61D**	2,3,5,7	synonymous SNV	7	6.862745098
ORF1a;ORF1ab;nsp4	C8986T	**D144D**	1,2,3,4,5,6,7,10	synonymous SNV		
ORF1a;ORF1ab;nsp4	G9053T	**V167L**	1,2,3,4,5,6,7,10	nonsynonymous SNV		
ORF1a;ORF1ab;nsp4	C9474T	**A307V**	1	nonsynonymous SNV		
ORF1a;ORF1ab;nsp4	C9857T	**L435L**	8,9	synonymous SNV		
ORF1a;ORF1ab;nsp4	C9891T	**A446V**	8,9	nonsynonymous SNV		
ORF1a;ORF1ab;nsp4	C10029T	**T492I**	1,2,3,4,5,6,7,10	nonsynonymous SNV		
ORF1a;ORF1ab;nsp5	C10232T	**R60C**	10	nonsynonymous SNV	2	1.960784314

ORF1a;ORF1ab;nsp5	C10279T	**L75L**	5	synonymous SNV		
ORF1a;ORF1ab;nsp6	A11201G	**T77A**	1,2,3,4,5,6,7,10	nonsynonymous SNV	5	4.901960784
ORF1a;ORF1ab;nsp6	A11332G	**V120V**	1,2,3,4,5,6,7,10	synonymous SNV		
ORF1a;ORF1ab;nsp6	T11418C	**V149A**	8,9	nonsynonymous SNV		
ORF1a;ORF1ab;nsp6	C11514T	**T181I**	8,9	nonsynonymous SNV		
ORF1a;ORF1ab;nsp6	C11671T	**R233R**	8,9	synonymous SNV		
ORF1a;ORF1ab;nsp7	NA					
ORF1a;ORF1ab;nsp8	C12676T	**V195V**	7	synonymous SNV	1	0.980392157
ORF1a;ORF1ab;nsp9	C13019T	**L112L**	8,9	synonymous SNV	1	0.980392157
ORF1a;ORF1ab;nsp10	C13168T	**H48H**	10	synonymous SNV	1	0.980392157
ORF1ab;nsp12	A13620G	**D60D**	10	synonymous SNV	10	9.803921569
ORF1ab;nsp12	A13748G	**K103R**	1,4,6	nonsynonymous SNV		
ORF1ab;nsp12	G14118T	**T226T**	4	synonymous SNV		
ORF1ab;nsp12	A14246G	**D269G**	8,9	nonsynonymous SNV		
ORF1ab;nsp12	C14407T	**P323S**	10	nonsynonymous SNV		
ORF1ab;nsp12	C14408T	**P323L**	all 10 samples	nonsynonymous SNV		
ORF1ab;nsp12	C14599T	**L387L**	4	synonymous SNV		
ORF1ab;nsp12	T14769C	**A443A**	2	synonymous SNV		
ORF1ab;nsp12	C14805T	**Y455Y**	2,3,5,7	synonymous SNV		
ORF1ab;nsp12	G15451A	**G671S**	all 10 samples	nonsynonymous SNV		
ORF1ab;nsp13	C16466T	**P5401L**	all 10 samples	nonsynonymous SNV	2	1.960784314
ORF1ab;nsp13	C16726T	**H5488Y**	4	nonsynonymous SNV		
ORF1ab;nsp14	A19008C	**A6248A**	8,9	synonymous SNV	2	1.960784314
ORF1ab;nsp14	C19220T	**A6319V**	1,2,3,4,5,6,7,10	nonsynonymous SNV		
ORF1ab;nsp15	G20464T	**D6734Y**	7	nonsynonymous SNV	1	0.980392157
ORF1ab;nsp16	G20808A	**L6848L**	6	synonymous SNV	2	1.960784314
ORF1ab;nsp16	G21255C	**A6997A**	1,4,6	synonymous SNV		
S	o	**T19R**	all 10 samples	nonsynonymous SNV	22	21.56862745
S	C21622T	**T20T**	10	synonymous SNV		
S	A21647G	**T29A**	2,3,5,7	nonsynonymous SNV		
S	C21721T	**D53D**	1,4,6	synonymous SNV		
S	G21800T	**D80Y**	4	nonsynonymous SNV		
S	C21811T	**V83V**	3	synonymous SNV		
S	C21846T	**T95I**	1,4,6,19	nonsynonymous SNV		
S	G21987A	**G142D**	all 10 samples	nonsynonymous SNV		
S	G22021T	**M153I**	10	nonsynonymous SNV		
S	AGTTCA22029.	**156_158del**	all 10 samples	nonframeshift deletion		
S	C22227T	**A222V**	8,9	nonsynonymous SNV		

S	C22264T	**N234N**	5	synonymous SNV		
S	C22311T	**T250I**	2,3,5,7	nonsynonymous SNV		
S	C22858T	**C432C**	3	synonymous SNV		
S	T22917G	**L452R**	all 10 samples	nonsynonymous SNV		
S	C22995A	**T478K**	all 10 samples	nonsynonymous SNV		
S	A23403G	**D614G**	all 10 samples	nonsynonymous SNV		
S	C23557T	**P665P**	10	synonymous SNV		
S	C23604G	**P681R**	all 10 samples	nonsynonymous SNV		
S	A23848G	**Q762Q**	8,9	synonymous SNV		
S	G24410A	**D950N**	all 10 samples	nonsynonymous SNV		
S	C25047T	**P1162L**	2	nonsynonymous SNV		
ORF3a	C25469T	**S26L**	all 10 samples	nonsynonymous SNV	4	3.921568627
ORF3a	C25584T	**T64T**	2,3,5,7	synonymous SNV		
ORF3a	C25667T	**S92L**	4	nonsynonymous SNV		
ORF3a	G25996T	**V202L**	10	nonsynonymous SNV		
M	C26684G	**L54L**	7	synonymous SNV	2	1.960784314
M	T26767C	**I82T**	all 10 samples	nonsynonymous SNV		
ORF6	C27371T	**P57L**	8,9	nonsynonymous SNV	1	0.980392157
ORF7a	G27461T	**C23F**	10	nonsynonymous SNV	4	3.921568627
ORF7a	C27476T	**T28I**	10	nonsynonymous SNV		
ORF7a	T27638C	**V82A**	all 10 samples	nonsynonymous SNV		
ORF7a	C27752T	**T120I**	all 10 samples	nonsynonymous SNV		
ORF7b	C27874T	**T40I**	1,2,3,4,5,6,7,10	nonsynonymous SNV	1	0.980392157
ORF8	GATTTC28248.	**119_120del**	all 10 samples	nonframeshift deletion	1	0.980392157
N	A28461G	**D63G**	all 10 samples	nonsynonymous SNV	7	6.862745098
N	C28677T	**T135I**	10	nonsynonymous SNV		
N	G28881T	**R203M**	all 10 samples	nonsynonymous SNV		
N	G28916T	**G215C**	1,2,3,4,5,6,7,10	nonsynonymous SNV		
N	G28936T	**L221F**	7	nonsynonymous SNV		
N	C29284T	**I337I**	2	synonymous SNV		
N	G29402T	**D377Y**	all 10 samples	nonsynonymous SNV		
ORF10	A29567G	**I4V**	4	nonsynonymous SNV	1	0.980392157
3'UTR	A29706G	**NA**	3	Downstream	3	2.941176471
3'UTR	G29742T	**NA**	all 10 samples	Downstream		
3'UTR	A29767C	**NA**	3	Downstream		

Total		102	

UTR: Un-translated region

NA: Not available

SNV: Single nucleotide variation

AA: Amino acids

Supplementary Table S2. Full genomic annotations of 2 Duhok isolates from the fifth wave of the SARS-CoV-2 pandemic

Genomic region	Nucleotide variation	AA change	Isolates	Exonic Function	Mutation per region	% of tatotal muattions
5'UTR	C44T	.		upstream	2	2.5
5'UTR	C241T	.		upstream		
ORF1a;ORF1ab;nsp1	C313T	L16L	exonic	synonymous SNV	2	2.5
ORF1a;ORF1ab;nsp1	T670G	S135R	exonic	nonsynonymous SNV		
ORF1a;ORF1ab;nsp2	C1627T	L274L	exonic	synonymous SNV	1	1.25
ORF1a;ORF1ab;nsp3	C2790T	T24I	exonic	nonsynonymous SNV	4	5
ORF1a;ORF1ab;nsp3	C3037T	F106F	exonic	synonymous SNV		
ORF1a;ORF1ab;nsp3	G4184A	G489S	exonic	nonsynonymous SNV		
ORF1a;ORF1ab;nsp3	C4321T	A534A	exonic	synonymous SNV		
ORF1a;ORF1ab;nsp4	C9344T	L264F	exonic	nonsynonymous SNV	5	6.25
ORF1a;ORF1ab;nsp4	A9424G	V290V	exonic	synonymous SNV		
ORF1a;ORF1ab;nsp4	C9534T	T327I	exonic	nonsynonymous SNV		
ORF1a;ORF1ab;nsp4	T9574C	G340G	exonic	synonymous SNV		
ORF1a;ORF1ab;nsp4	C10029T	T492I	exonic	nonsynonymous SNV		
ORF1a;ORF1ab;nsp5	C10198T	D48D	exonic	synonymous SNV	3	3.75
ORF1a;ORF1ab;nsp5	G10447A	R131R	exonic	synonymous SNV		
ORF1a;ORF1ab;nsp5	C10449A	P132H	exonic	nonsynonymous SNV		
ORF1a;ORF1ab;nsp6	TCTGGTTTT	106_108del	exonic	nonframeshift deletion	2	2.5
ORF1a;ORF1ab;nsp6	C11396	L142F	exonic	nonsynonymous SNV		
ORF1a;ORF1ab;nsp8	G12160A	E23E	exonic	synonymous SNV	2	2.5
ORF1a;ORF1ab;nsp8	G12310A	Q73Q	exonic	synonymous SNV		
ORF1a;ORF1ab;nsp9	C12880T	I65I	exonic	synonymous SNV	1	1.25
ORF1ab;nsp12	C14408T	P323L	exonic	nonsynonymous SNV	2	2.5
ORF1ab;nsp12	C15714T	L758L	exonic	synonymous SNV		
ORF1ab;nsp13	C16616A	T5451N	exonic	nonsynonymous SNV	2	2.5
ORF1ab;nsp13	C17410T	R5716C	exonic	nonsynonymous SNV		
ORF1ab;nsp14	A18163G	I5967V	exonic	nonsynonymous SNV	1	1.25
ORF1ab;nsp15	C19955T	T6564I	exonic	nonsynonymous SNV	2	2.5
ORF1ab;nsp15	A20055G	E6597E	exonic	synonymous SNV		
S	C21618T	T19I	exonic	nonsynonymous SNV	32	39.02439024
S	TACCCCCTG	24_27del	exonic	nonframeshift deletion		
S	TACATG	68_70del	exonic	nonframeshift deletion		
S	G21987A	G142D	exonic	nonsynonymous		

				SNV		
S	T22200G	V213G	exonic	nonsynonymous SNV		
S	G22578A	G339D	exonic	nonsynonymous SNV		
S	C22674T	S371F	exonic	nonsynonymous SNV		
S	T22679C	S373P	exonic	nonsynonymous SNV		
S	C22686T	S375F	exonic	nonsynonymous SNV		
S	A22688G	T376A	exonic	nonsynonymous SNV		
S	G22775A	D405N	exonic	nonsynonymous SNV		
S	A22786C	R408S	exonic	nonsynonymous SNV		
S	G22813T	K417N	exonic	nonsynonymous SNV		
S	T22882G	N440K	exonic	nonsynonymous SNV		
S	T22917G	L452R	exonic	nonsynonymous SNV		
S	G22992A	S477N	exonic	nonsynonymous SNV		
S	C22995A	T478K	exonic	nonsynonymous SNV		
S	A23013C	E484A	exonic	nonsynonymous SNV		
S	T23018G	F486V	exonic	nonsynonymous SNV		
S	T23042C	S494P	exonic	nonsynonymous SNV		
S	A23055G	Q498R	exonic	nonsynonymous SNV		
S	A23063T	N501Y	exonic	nonsynonymous SNV		
S	T23075C	Y505H	exonic	nonsynonymous SNV		
S	A23403G	D614G	exonic	nonsynonymous SNV		
S	C23525T	H655Y	exonic	nonsynonymous SNV		
S	T23599G	N679K	exonic	nonsynonymous SNV		
S	C23604A	P681H	exonic	nonsynonymous SNV		
S	C23854A	N764K	exonic	nonsynonymous SNV		
S	G23948T	D796Y	exonic	nonsynonymous SNV		
S	A24424T	Q954H	exonic	nonsynonymous SNV		
S	T24469A	N969K	exonic	nonsynonymous SNV		
S	C2500T	D1146D	exonic	synonymous SNV		
ORF3a	C25584T	T64T	exonic	synonymous SNV	4	5
ORF3a	C25896T	V168V	exonic	synonymous SNV		

ORF3a	C26060T	T223I	exonic	nonsynonymous SNV		
ORF3a	G26109T	E239D	exonic	nonsynonymous SNV		
E	C26270T	T9I	exonic	nonsynonymous SNV	1	1.25
M	G26529A	D3N	exonic	nonsynonymous SNV	4	5
M	C26577G	Q19E	exonic	nonsynonymous SNV		
M	G26709A	A63T	exonic	nonsynonymous SNV		
M	C27012T	L164L	exonic	synonymous SNV		
ORF7a	C27513T	Y40Y	exonic	synonymous SNV	2	2.5
ORF7b	C27807T	L18L	exonic	synonymous SNV		
N	C28311T	P13L	exonic	nonsynonymous SNV	7	8.75
N	A28330G	G19G	exonic	synonymous SNV		
N	GAGAACGCA	30_33del	exonic	nonframeshift deletion		
N	G28881A	R203K	exonic	nonsynonymous SNV		
N	G28882A	R203R	exonic	synonymous SNV		
N	G28883C	G204R	exonic	nonsynonymous SNV		
N	A29510C	S413R	exonic	nonsynonymous SNV		
N;ORF10	GAGGCCACGCGGAGTACGATCGAGTG	.		downstream	1	1.25
					80	

UTR: Un-translated region

NA: Not available

SNV: Single nucleotide variation

AA: Amino acids
